# A Bio-Inspired Arched Foot with Individual Toe Joints and Plantar Fascia

**DOI:** 10.3390/biomimetics8060455

**Published:** 2023-09-26

**Authors:** Stuart Burgess, Alex Beeston, Joshua Carr, Kallia Siempou, Maya Simmonds, Yasmin Zanker

**Affiliations:** Bristol Robotics Laboratory, School of Electrical, Electronic & Mechanical Engineering, Bristol University, Bristol BS8 1QU, UKpi18863@bristol.ac.uk (Y.Z.)

**Keywords:** robotic foot, foot prosthetic, MTP joints, arch stiffness, windlass mechanism, plantarflexion

## Abstract

This paper presents the design and testing of an arched foot with several biomimetic features, including five individual MTP (toe) joints, four individual midfoot joints, and plantar fascia. The creation of a triple-arched foot represents a step further in bio-inspired design compared to other published designs. The arched structure creates flexibility that is similar to human feet with a vertical deflection of up to 12 mm. The individual toe joints enable abduction–adduction in the forefoot and therefore a natural pronation motion. Adult female bone data was obtained and converted into a CAD model to accurately identify the location of bones, joints, and arches. An analytical model is presented that gives the relationship between the vertical stiffness and horizontal stiffness of the longitudinal arches and therefore allows the optimization of stiffness elements. Experimental tests have demonstrated a vertical arch stiffness of 76 N/mm which is similar to adult human feet. The range of movement of the foot is similar to human feet with the following values: dorsi-plantarflexion (28°/37°), inversion-eversion (30°/15°), and abduction–adduction (30°/39°). Tests have also demonstrated a three-point contact with the ground that is similar to human feet.

## 1. Introduction

### 1.1. Background

This paper presents the design and testing of a flexible arched foot with several biomimetic features, including five individual metatarsophalangeal (MTP) (toe) joints, four individual midfoot joints, and plantar fascia. The creation of a triple-arched foot with a medial arch, lateral arch, and transverse arch represents a step further in bio-inspired design compared to other published designs. The foot is intended for use as a robotic or prosthetic foot either as a passive or actuated system. This paper deals with a passive design.

Flexible arches and MTP (toe) joints are known to have advantages such as enabling walking on uneven ground [1], shock absorption [2], and energy storage and release [3]. Some researchers have recommended that future robotic and prosthetic feet should incorporate more design features of the human foot to achieve high performance [4,5]. The metatarsophalangeal (toe) joints play an important role in locomotion on uneven surfaces, especially during ground push-off [6,7,8,9]. Robotic and prosthetic feet with toe joints have also been shown to improve locomotion efficiency [10,11]. Studies have shown that storing elastic energy is an important component of walking [12], showing that flexible elements can be advantageous in robotic feet. Some studies have shown that most amputees express a preference for prosthetics that copy human biomechanics because of the natural feel that is produced [11,13]. There is also evidence that people prefer humanoid robots to have human-like movements [14]. Having robotic feet with natural movements and human abilities therefore can be desirable.

Traditional robotic feet have been typically flat and rigid with a plantar-dorsiflexion joint or universal joint at the ankle. Such designs typically only allow short shuffling strides that are inefficient and cannot cope with uneven surfaces [15]. In recent years there has been some progress in designing flexible and arched feet that can produce a longer and more human-like gait. One example is the DURUS robot, which has an arched foot with specific walking abilities including heel-strike, toe-strike, and toe push-off [16,17]. Springs between the ankle and feet (similar to elastic tendons) allow for a walking gait that stores mechanical energy from a heel strike which is then reclaimed as the foot lifts off the ground. Another example of a flexible arched robotic foot is that of Kwon et al. [18], who designed a foot with two contact points on the heel and three spring-loaded contact points on the ball of the foot. Pressure sensors are used to control the position of the foot. A foot that emulates the rotational motions of the human subtalar and oblique midtarsal joints has been produced by Chen et al. [19]. This design is driven by a bevel-geared mechanism that compensates for the yaw moment created in bipedal walking. The motions generate a yaw moment at touch-down and may contribute to suppressing the yaw rotation of the whole body. The use of compliant legs for traversing uneven ground has also been used on multi-legged robots [20].

Traditional prosthetic feet have also tended to be based on a stiff lever and there has been limited progress in implementing human design features into prosthetic feet [21,22]. There have been some bio-inspired foot prosthetics that use compliant elements [23,24,25]. A prosthetic foot with a midfoot joint and MTP joint called the K3 promoter was published in 2010 [26]. The midfoot joint in the K3 effectively creates a flexible longitudinal arch, although there are no individual toes and hence limited forefoot abduction–adduction. Since 2020, prostheses with MTP joints have recently entered the commercial market (e.g., Ottobock Meridium, ST&G ToeFlex). These prosthetics are advertised as having a ‘natural feel’.

### 1.2. Relevant Foot Anatomy

**Figure 1:**
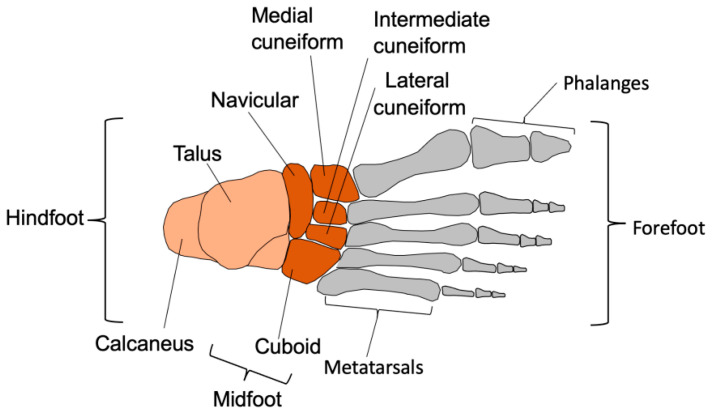
Bones of the human foot.

As shown in Figure 1, the human foot can be considered to have three distinct parts: the hindfoot, midfoot, and forefoot. There are 2 bones in the hindfoot, 5 bones in the midfoot, and 19 bones in the forefoot. The human foot has a multi-configurable system that allows it to have optimal stiffness at different stages of the gait cycle [27]. During the landing phase, the foot needs to be flexible and shock-absorbing, but during the push-off phase, the foot needs to be a stiff lever. Fine-tuning of stiffness occurs due to active mechanisms such as intrinsic muscles [28] but also passive mechanisms.

**Figure 2:**
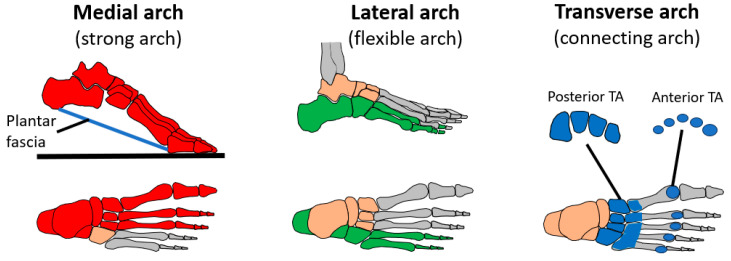
Three arches of the human foot.

The bones of the human foot form three arched structures as shown in Figure 2. The longitudinal arches, especially the medial arch, form a stiff lever for walking and running. The arches also form flexible structures for absorbing shock loads [1]. The transverse arch acts to stiffen the longitudinal arches during push-off [29] and also stores energy during walking and running [30]. The transverse arch can be considered to have two main sections: the posterior transverse arch at the base of the metatarsal bones and the anterior transverse arch at the heads of the metatarsal bones. The anterior transverse arch is considered complete because the heads of the first and fifth metatarsals both come into contact with the ground and form the two ends of the arch. The foot has a plantar fascia ligament under the foot that automatically stiffens the longitudinal arches during walking and running as the MTP (toe) joints rotate to produce a windlass-type mechanism [31].

**Figure 3:**
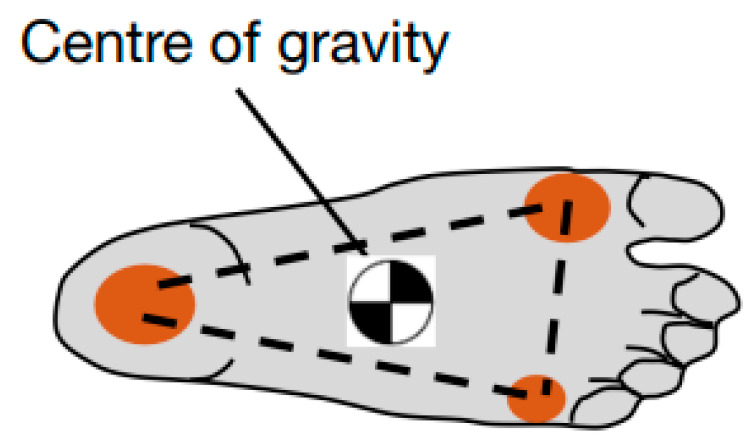
Three point-contact from three arches in the foot.

The three arches of the foot combine to create three-point contact with the ground. The ball of the foot has two contact points from the anterior transverse arch as well as the anterior ends of the longitudinal arches. The heel has a contact point at the posterior end of the longitudinal arches. The three-point contact is important because it greatly aids balance. For example, it is possible to stand on one leg by placing the center of gravity of the body inside the three contact points as shown in Figure 3 [32].

### 1.3. Main Joints and Movements of the Human Foot

**Figure 4:**
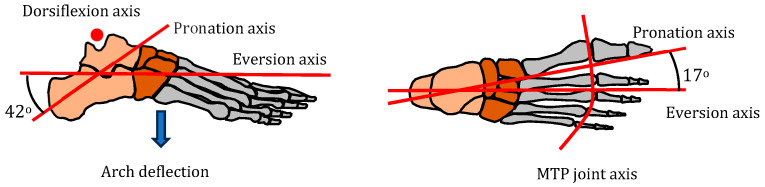
Foot axes of rotation (pronation axis from [33]).

The five main biomechanical movements of the human foot are shown in Figure 4 and summarized in Table 1.

### 1.4. Design Goals

The main aim of our foot design was to emulate the three arches and enable the five main biomechanical movements of the human foot shown in Table 1. Another biomechanical goal was to enable three-point contact with the ground. A manufacturing objective was to use 3D printing technology for production because this has important advantages such as speed of production, reduced cost, and customization [34,35,36]. The geometry of the foot is based on the bones of an adult female because of the lack of representation of females in medical research [37,38]. Another objective was to meet ISO prosthetics standards for strength tests [39].

## 2. Materials and Methods

Human bone data was obtained in order to identify the proportions of the bio-inspired foot as accurately as possible, such as the location of joints and arches. A 3D scan of the bones in a female foot was sourced from Embodi3D, a biomedical 3D printing community hub. Three-dimensional printing of bones from scanned data is now an established technique [40]. The CT scan used was that of a 60-year-old female. The file was imported into the Fusion 360 design workspace and was rescaled to a length of 246 mm, which correlates the model to the average UK female foot size of 6 [41].

**Figure 5:**
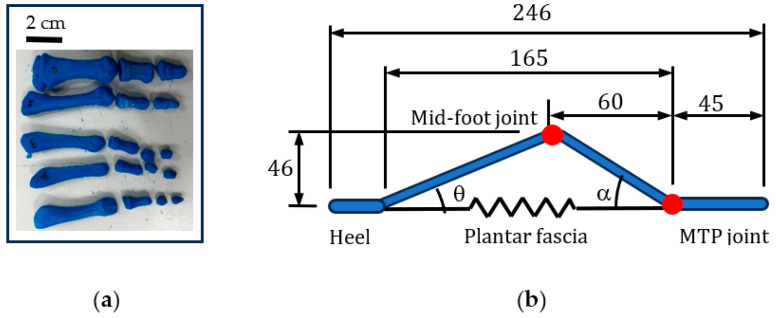
An adult female foot was used to determine key geometric points. (**a**) Three-dimensional printed bones (**b**) Geometry of middle toe.

The file from Embodi3D was accompanied by noise in the form of disconnected facets floating around the model which were removed. In some bones such as the metatarsals, there were small areas of missing geometries that required interpolating. These bones were imported into Siemens NX (NX 2212) software which is capable of applying reverse engineering techniques such as spline fitting. This technique involves creating boundary curves around the geometry using control points and selecting the appropriate degree and segments so that the splines fit as closely to the geometry as possible. A high density of facets was utilized in the rebuild to ensure that detail was retained. To validate the computer-aided design (CAD) model, the female foot bones were 3D printed as shown in Figure 5a. The CAD model was used to extract key dimensions such as the location of the mid-joints and MTP joints for each toe. An example of the middle toe is shown in Figure 5b.

A vertical arch stiffness of 82 N/mm was chosen as a design goal. We found three examples in the literature of adult vertical arch stiffness measurements between 76 and 88 N/mm [42,43,44]. An ankle stiffness of 600 Nm/rad for eversion-inversion and dorsiflexion-plantarflexion was chosen as a design goal as field studies have shown this level to be within the range of popular choices for users [45,46].

## 3. Design Solution

**Figure 6:**
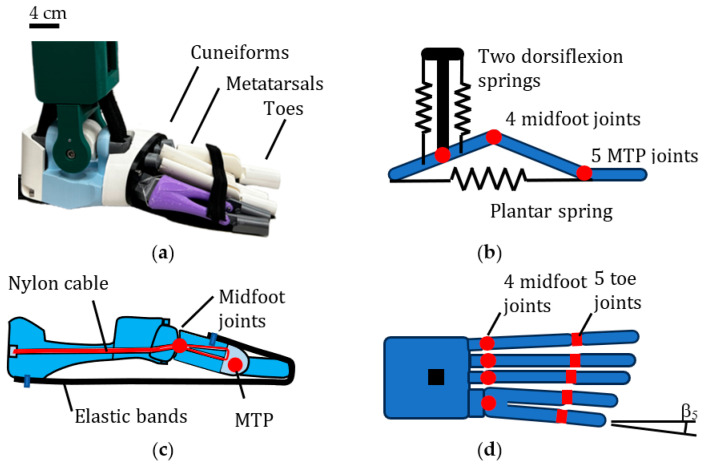
Bio-inspired foot design. (**a**) Prototype (no plantar fascia). (**b**) Schematic side view. (**c**) Section showing nylon cable (**d**) Plan view of foot.

Figure 6a shows a prototype model of the bio-inspired foot. The design replicates (in a simplified form) the three arches of the human foot. There are five (single component) toes that are attached via five MTP joints to five metacarpal bones. However, for practical reasons, the two smallest metacarpal bones merge into one where they meet the cuboid bone of the midfoot.

There are four midfoot joints between the metacarpals and the midfoot bones (three cuneiform bones and the cuboid bone). These four joints form the midfoot joints of the foot, as shown in Figure 6b–d. There is no navicular bone. The four midfoot bones are rigidly constrained by an outer ring and do not form a flexible arch but do provide an interface for the metacarpals. The plantar fascia consists of 10 elastic bands (2 per toe).

Each toe is simplified to one bone, and this is attached to a metacarpal by a plane bearing to allow flexion. The metacarpal bones are attached to the midfoot bones via a spherical interface and nylon cable, as shown in Figure 6c. A commercial nylon cable of 0.42 mm diameter was chosen to perform the ligament function of joining the bones together. The reason for choosing a slender cable was twofold: firstly, to allow ease of bending, and secondly, to allow multiple strands to be used in order to optimize stiffness and strength. A total of 24 strands were used (6 per toe). Multiple nylon strands in robotic joints have been used previously in a bio-inspired knee joint [47]. The ligaments are tightened with Allen keys via a mechanism at the heel of the foot. The use of a spherical joint allows for abduction–adduction of the forefoot as well as vertical deflection of the longitudinal arches. The abduction/adduction movement at the midfoot enables pronation similar to the human foot.

At the ankle joint, there is a universal joint with two crossed bearings in a gimbal arrangement, as shown in Figure 6a. Stiffness for dorsi-plantarflexion is created via two commercial elasticated cords with a braided sheath of 1.2 cm diameter (Figure 6a,b). Stiffness for inversion/eversion is created by restraining each elastic cord with two pairs of aluminum pins close to the axis of rotation. The weight of the bio-inspired foot is 1.25 kg.

The elastic bands are attached to the solid components by a hybrid connection consisting of an adhesive and screw/washer. Different connection types were tested, including adhesive-only, adhesive with dowel, and adhesive with screw/washer, with the latter giving the strongest joint. The elasticated cords used for the ankle stiffness elements were held using a screwed clamping system. Because of the horizontal displacement of the foot, it is preferable that the foot is used with a shoe in order to have control over the friction at the base of the arch.

Polyethylene terephthalate glycol (PETG) was chosen for the rigid components of the foot because it is a commonly used polymeric material in the healthcare sector, as well as suited to 3D printing. The material is good for medical applications mainly due to its biocompatibility, high uniformity, mechanical strength, and resistance against chemicals and abrasion. It also has potential for antibacterial properties [48].

An advantage of 3D printing is that microstructure can be added using the infill density option. Microstructure brings down the effective density of the material by creating air voids. The solid parts of the foot were 3D printed with a triangular infill structure with an infill density of 50%, thus reducing the effective elastic modulus. Microstructure is efficient from a structural viewpoint because beams can be made with a larger depth for a given amount of material and therefore have a higher second moment of area. This increases both the specific stiffness and specific strength [49]. Three-dimensional printing was carried out on an Ultimaker S3 which has a default print speed of 70 mm/s. The print time on a single 3D printer was 76 h and 14 min, but this time can be reduced using multiple or faster machines. For example, using two 3D printers with a 250 mm/s print speed would give a production time of around 10 h.

A commercial elastic band (used in clothing) of cross section 12.8 mm × 1 mm was chosen to replicate the plantar fascia (using multiple bands). It comprises thin rubber strands woven together in a fabric sheath of composition 30% polypropylene fabric and 70% latex. Commercial nylon cable was chosen for a ligament role in holding bones together. Commercial elasticated cord of the type used in bungee jumping was used for the ankle stiffness elements.

## 4. Modeling

Having selected a system of joints to replicate the three arches of the human foot, the next stage of design was to optimize stiffness levels to match the human foot and/or preferred stiffness levels in foot prosthetics.

### 4.1. Modeling Arch Stiffness

**Figure 7:**
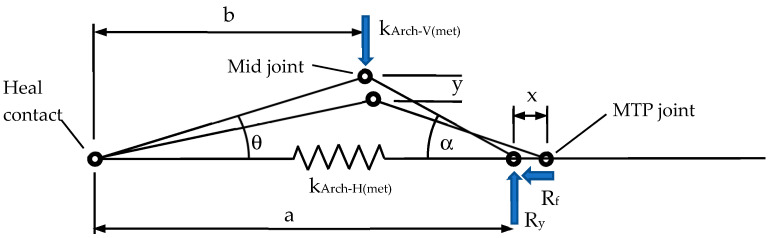
Model of each metatarsal arch as a mechanism.

In order to achieve a similar arch stiffness to the human foot, it was necessary to select suitable stiffness elements for the elastic bands (plantar fascia) and nylon strands (ligaments). Since the elastic bands and nylon strands determined the lateral stiffness of the arch, it was necessary to find an approximate relationship between the vertical arch stiffness *k_Arch − V_* and the horizontal arch stiffness *k_Arch − H_*. To determine this relationship, a simplified model of each metatarsal arch as a mechanism was constructed, as shown in Figure 7.

When the arch is deflected downwards by distance *y*, the length of an individual metacarpal arch increases by an amount *x* as shown in Figure 7. Account has to be taken of a friction force that adds resistance to the stretching of the plantar fascia spring. Account also has to be taken of the changing stiffness of the arch as it deflects.

The vertical and horizontal stiffness of an individual metacarpal arch is defined as follows:

(1) $$k_{Arch-V(met)}=\frac{∆P}{∆y}$$

(2) $$k_{Arch-H(met)}=\frac{T}{x}$$

where *P* is the load at the mid joint and *T* is the load in the horizontal spring(s).

For small deflections (e.g., at the start of deformation) of an individual metacarpal, the tension in the horizontal spring is given by:

(3) $$T=\frac{Pb}{a}\;\left(cot(\alpha )-\mu \right)$$

where *μ* is the coefficient of friction which was assumed to be 0.3 for the polypropylene sheath [50].

The extension of the plantar fascia for the metacarpal arch is approximately given by:

(4) $$x\approx y\;\left(tan\left(\alpha \right)+tan\left(\theta \right)\right)$$

Therefore, an approximate expression for the vertical stiffness of an individual metacarpal arch is given by:

(5) $$k_{Arch-V(met)}\approx \;k_{Arch-H\left(met\right)}\frac{a\left(tan\left(\alpha \right)+tan\left(\theta \right)\right)}{b\left(cot\left(\alpha \right)-\mu \right)}$$

The relationship between *k_Arch − V(met)_* and *k_Arch − H(met)_* is therefore given by:

(6) $$k_{Arch-H(met)}={C_{1}k}_{Arch-V(met)}$$

where the constant *C*_1_ depends on the instantaneous geometry of the individual metacarpal arch.

The total horizontal stiffness of the foot is calculated by combining the stiffnesses of the five metacarpal arches. To achieve an average vertical stiffness of 82 N/mm (Section 2), the predicted total horizontal stiffness of the five toes is required to be *k_Arch − H_* = 117 N/mm.

The total horizontal stiffness of the foot is a function of the stiffness of the elastic bands and the nylon strands as follows:

(7) $$\begin{array}{c} k_{Arch-H}=k_{B1}cos\beta_{1}+k_{B2}cos\beta_{2}+k_{B3}cos\beta_{3}+k_{B4}cos\beta_{4}+k_{B5}cos\beta_{5}+{\;k}_{N1}cos\beta_{1}q_{1}+k_{N2}cos\beta_{2}q_{2}\\ \;\;\;\;\;\;\;\;\;\;\;\;\;\;\;\;\;\;\;\;\;\;\;\;\;\;+k_{N3}cos\beta_{3}q_{3}+k_{N4}cos\beta_{4}q_{4} \end{array}$$

where *k_B_* is the stiffness of two elastic bands, *k_N_* is the stiffness of six nylon strands, *b* is the angle of each toe as defined in Figure 6, and *q_(n)_* is a constant that takes into account the difference in radius of the nylon strands compared to the elastic bands. The stiffness of the elastic bands and nylon strands is given by *AE/L* where *A* is the cross-sectional area, *E* is the Young’s modulus, and *L* is the length of each stiffness element. The cross-sectional area of the elastic bands and nylon strands was chosen to produce the required horizontal arch stiffness of the foot.

### 4.2. Modeling Ankle Stiffness

The stiffness of the ankle in dorsiflexion is given by:

(8) $$k_{Ankle-flexion}=\frac{EA}{L}r^{2}$$

where *E* is the Young’s modulus, *A* is the cross-sectional area, *L* is the length of unrestrained cord, and *r* is the radius at which the elastic cord is acting. From the above equation, the length and location of the elastic cord were selected to give the desired stiffness of 600 Nm/rad (Section 2).

### 4.3. Modeling Windlass Mechanism

When pushing off in walking and running, the longitudinal arch is stiffened by a passive mechanism of plantar fascia tightening (windlass effect) [51]. When the toes turn upwards at the MTP joints, the load in the plantar fascia (elastic band) is increased as the fascia is stretched around the base of the toes. As the toes move upwards by an angle *q*, the band is extended by a distance of *qr* where *r* is the radius of the tarsal bone. The increase in tension DT is given by:

(9) $$∆T=k_{Arch-H}\theta r$$

For a rotation of 45 degrees at the MTP joints, the increase in tension is approximately 49 N which significantly stiffens the arch.

## 5. Results

### 5.1. Stiffness and Loading Tests

**Figure 8:**
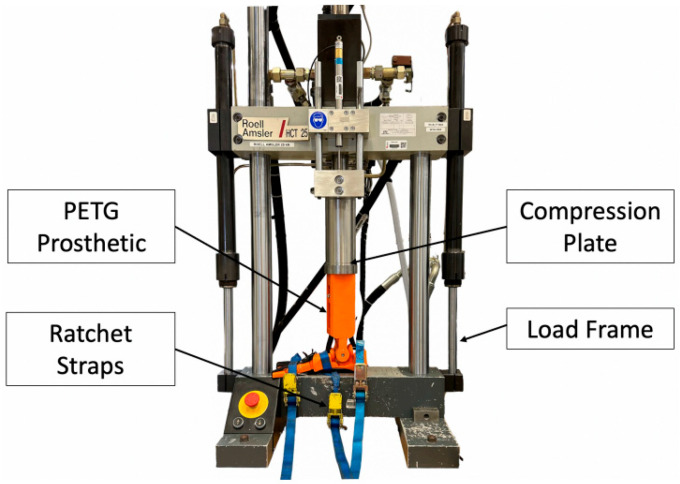
Stiffness test on the bio-inspired foot.

**Figure 9:**
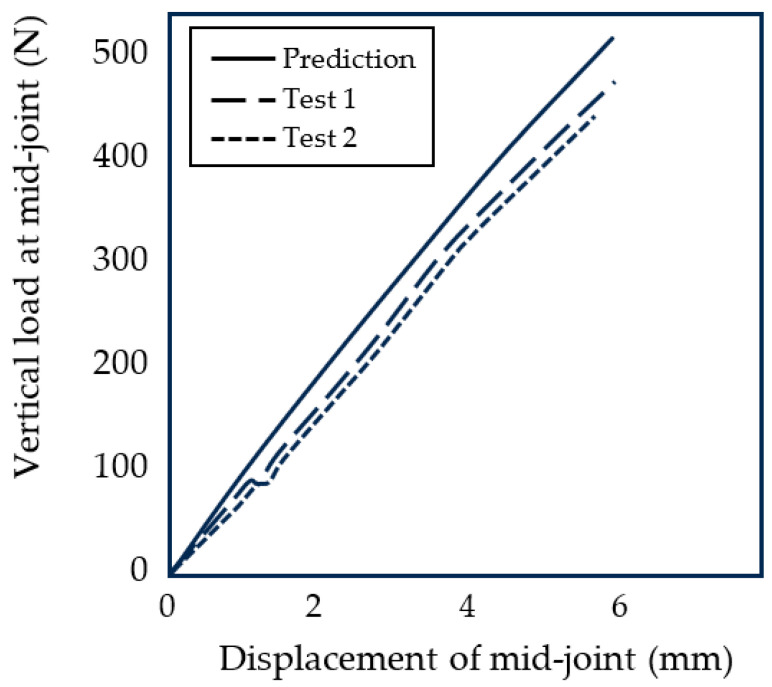
Vertical stiffness of the arch at the midfoot joint.

The stiffness test was performed on a 25 kN Instron 8872 loading machine, as shown in Figure 8. The compression test was programmed on the Instron WaveMatrix v2 which applied a force to the leg which increased from 0 to 1800 N at a rate of 10 N/s. Two test results are shown in Figure 9 and compared with the theoretical predictions (Equation (6)). The stiffness of the foot was measured to have an average stiffness of approximately 76 N/mm which is at the lower end of the target range of 76–88 N/mm (Section 2). 

The 25 kN Instron 8872 was also used for the proof load tests. The foot passed the proof test of the highest ISO loading level P8 for prosthetic feet of 5700 N [39] as well as the ultimate static test force at level P3 of 3220 N [39]. It was found that an infill density of 50% for the components was adequate for meeting the ISO standards.

### 5.2. Range of Movement Tests

**Figure 10:**
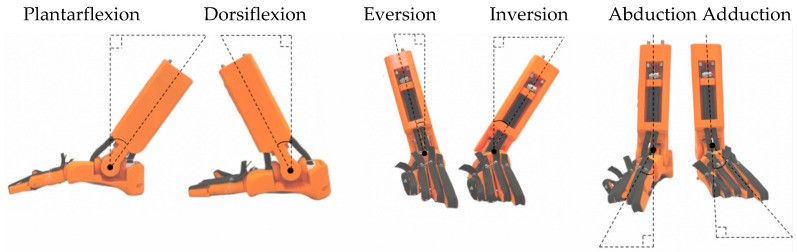
Extreme positions of the bio-inspired foot (all right foot).

The range of movement (ROM) of the bio-inspired foot was tested, as shown in Figure 10 and Table 2. The tests involved moving the foot to maximum positions and taking an image for analysis. The range of movement is generally similar to a healthy human foot although plantarflexion is lower for the bio-inspired foot by around 25%. However, this should not significantly hinder activities such as walking. The prosthetic has a total ROM in the sagittal plane of 65 degrees which is just within the normal range of motion for healthy adults of 65–75 [33].

### 5.3. Ground Contact Test

**Figure 11:**
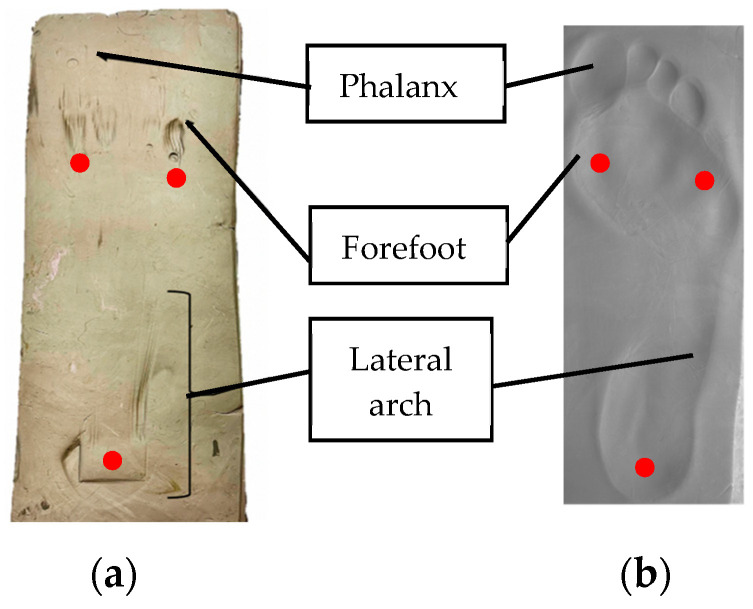
Contact patch of the bio-inspired foot and human foot (right foot). (**a**) Bioinspired foot (**b**) Human foot.

A plasticene imprint test was carried out on the bioinspired foot to demonstrate three-point contact with the ground. A load of 55 kg was applied to the bioinspired foot which corresponds to the recommended weight for a female of average height in the UK. The load was applied through the leg. The resulting imprint is shown in Figure 11 together with the imprint of the foot of a 55 kg female. The results show that, like the human foot, the lateral arch of the bioinspired foot touches the ground due to the deflection of that arch. As with the human foot, the bio-inspired foot has three-point contact with the ground (shown by the red dots) including a contact point at the base of the big toe and base of the little toe.

## 6. Discussion

The longitudinal arch of the bio-inspired foot was demonstrated to have a similar vertical stiffness to the human foot with a gradual reduction in stiffness for increasing loading due to the flattening of the arch. There is broad agreement between the modeled foot stiffness and the measured stiffness values. The differences between the predicted and measured stiffness values (Figure 9) are likely to be due to the uncertainties in friction levels and material properties. The experimental results do not produce a smooth curve and have some discontinuities. There are several possible causes for this. First, the bottom contact points of the five metacarpal arches are not perfectly aligned with the ground and so do not all start to deform at exactly the same time. Secondly, there are friction effects in the spherical joints between the metacarpal bones and the mid-joint bones. Thirdly, there are stick–slip effects when the individual arches slide against the ground. As explained in Section 3, because of the horizontal displacement of the foot, it is preferable that the foot is used with a shoe in order to have control over the friction at the base of the arch. It is interesting to note that the vertical stiffness of a human foot will likewise be affected by the friction levels between the bottom of the foot and the contact surface.

Having separate medial and lateral arches in the bio-inspired foot means that the foot has the potential to land on the softer lateral arch and push off from the stiffer medial arch. It also means that there can be three-point contact through the three reaction points of the three arches, thus enabling balancing actions such as standing on one leg. The triple-arched structure also allows for human-like movements as well as the ability to cope with uneven surfaces. Having MTP joints enables a small degree of abduction of the toes which in turn enables true pronation when added to subtalar eversion and ankle dorsiflexion. 

**Figure 12:**
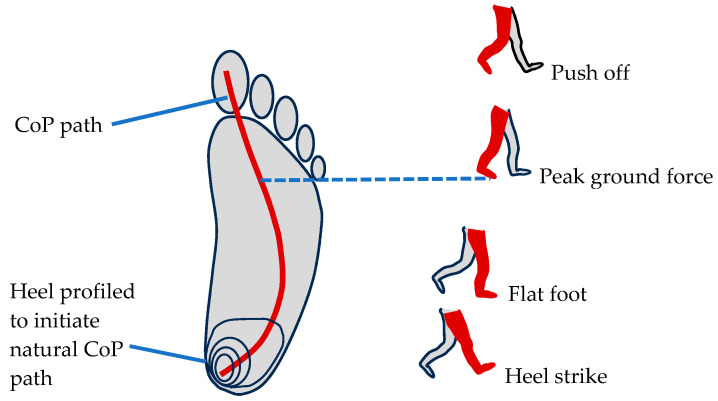
Profiled heel to initiate natural CoP pathway.

**Figure 13:**
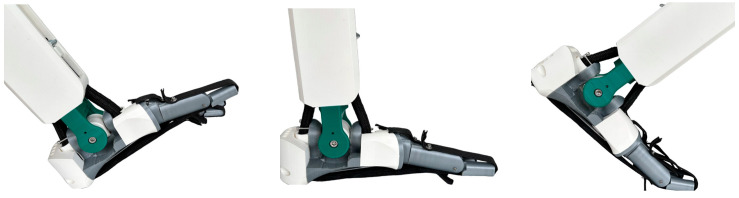
Bio-inspired foot during the gait cycle.

The focus of this study has been the optimization and testing of the static stiffness and strength of the bio-inspired foot. In the next step, we plan to optimize the design to replicate a center-of-pressure (CoP) pathway similar to the human foot. A typical center-of-pressure pathway starts on the medial side and then moves to the lateral side before returning to the medial side [54] as shown in Figure 12. The heel of the bio-inspired design has a profiled topography to initiate the first part of a natural CoP pathway, as shown in Figure 12. Optimization of the CoP is expected to be a complex task because there are many variables and degrees of freedom in the five toe arches. The variables include the geometry of each toe arch as well as the stiffness values of each individual joint. The modeling of the arch stiffness developed in this paper will help in the optimization process. The bio-inspired foot during the walking gait cycle is shown in Figure 13. Another objective for future designs is to optimize for reliability as the high number of joints can lead to potential weak points for both robotics and prosthetics.

## 7. Conclusions

The three arches of the human foot are key design features that optimize stiffness and contact with the ground. Copying the design of the human foot is very challenging because of the high number of joints and the difficulty in matching the properties of biological materials. However, in order to reproduce sophisticated functions such as walking on uneven ground or running, it is necessary to match key design features of the human foot. The implementation of three arches with individual toe and midfoot joints in our bio-inspired design represents a step further in bio-inspired design compared to other published designs. The foot has a similar vertical arch stiffness to the human foot and a similar range of movement. Three-dimensional printing was found to produce parts of aadequate strength and to enable customized parts. The bio-inspired foot presented here has potential use in robotics and prosthetics where human-like motion is desired.

## Figures and Tables

**Table 1:** Five movements of the human foot.

Movement	Joints Involved	Key Functions
Dorsiflexion Plantarflexion	Ankle (talocrural or talus-tibia) joint	Walking, running
Eversion Inversion	Subtalar (calcaneus-talus) joint and transverse tarsal joints	Cope with uneven ground
Pronation	Eversion, ankle dorsiflexion, and forefoot abduction	Shock absorption in running
Arch deflection	Rearfoot joints, midfoot joints, and metatarsal joints	Shock absorption
Toe dorsiflexion	MTP joints	Running

**Table 2:** Range of movements of the bio-inspired foot.

Movement	Human (Degrees)	Bio-Inspired Foot (Degrees)
Dorsiflexion [52]	20	28
Plantarflexion [52]	50	37
Inversion [52]	35	30
Eversion [52]	15	15
Abduction [53]	34	30
Adduction [53]	32	39

## Data Availability

No new data were created or analyzed in this study. Data sharing is not applicable to this article.
